# Aortic Valve Endocarditis in an Intravenous Drug User With Psychiatric History: A Diagnostic Challenge

**DOI:** 10.7759/cureus.80943

**Published:** 2025-03-21

**Authors:** Fnu Samaksh, Kesar Prajapati, Poornima Jaiswal Charpuria, Ma. Karen Lipana, Savi Mushiyev

**Affiliations:** 1 Internal Medicine, New York Medical College, Metropolitan Hospital Center, New York, USA; 2 Cardiology, New York Medical College, Metropolitan Hospital Center, New York, USA

**Keywords:** aortic valve disease, echocardiography - heart failure - valvular heart disease, infective endocarditis, intravenous drug use (ivdu), schizophrenia

## Abstract

Infective endocarditis (IE), a life-threatening cardiac infection, can present atypically, complicating diagnosis. We present a case of a 45-year-old male patient with schizophrenia and intravenous drug use (IVDU) who presented with suicidal ideation. Though afebrile, he exhibited tachycardia, bilateral pedal edema, bronchial breath sounds, and a diastolic murmur. Echocardiography identified an extensive aortic valve vegetation and a reduced ejection fraction. Despite negative resected valve cultures and Gram staining, emergency aortic valve replacement and left atrial appendage clipping were performed, followed by a six-week antibiotic course. This case underscores the diagnostic challenge of afebrile, culture-negative IE, likely due to prior antibiotics or fastidious organisms, particularly in high-risk populations like those with IVDU. Psychiatric presentations may obscure typical IE symptoms, necessitating heightened clinical suspicion and comprehensive evaluation, including echocardiography, even without classic signs like fever or leukocytosis. Timely intervention and individualized diagnostics are critical to improving outcomes in such complex cases.

## Introduction

Infective endocarditis (IE) is an endocardium infection involving native heart valves, prosthetic valves of the heart, or an implanted cardiac device [1]. It is a rare but life-threatening condition with a mortality of 15-20% in high-income countries [1]. Studies have shown increasing trends of IE in the world and the United States with annual incidence ranging from 3 to 10 per 100,000 persons in the United States; globally IE shows 1.58 million disability-adjusted life-years increases in IE-related healthcare expenditure [2].

Factors associated with IE are valvular disease, intravenous drug use (IVDU), cardiac surgery, poor dentition, and immunocompromised state [3]. The pathophysiology of IE begins with endothelial damage, which exposes subendothelial collagen and other matrix molecules. This exposure leads to the formation of a sterile thrombus composed of platelets and fibrin. Bacterial or fungal pathogens circulating in the bloodstream can adhere to this thrombus, leading to colonization and the formation of vegetation. These vegetations provide a protective environment for the pathogens, allowing them to proliferate and evade the host's immune response. The vegetation can become friable and embolized, leading to complications such as stroke, mycotic aneurysms, and systemic embolization. Additionally, the infection can cause valvular destruction, leading to severe regurgitation and heart failure [4,5].

Blood culture-negative infective endocarditis (BCNIE) constitutes approximately 30% of all IE cases with aortic (55%) and mitral (50%) valve affection posing a significant clinical challenge [6]. Diagnosing IE in those with IVDU presents unique challenges due to the often-atypical presentation and the high prevalence of right-sided IE, which may not exhibit classic peripheral stigmata. The diagnosis is further complicated by the social determinants of health, which contribute to higher mortality rates in low-income countries. These determinants include limited access to healthcare, poor living conditions, and higher rates of comorbidities [7,8].

This case report highlights the diagnostic challenges and management complexities of IE in a patient with IVDU, emphasizing the need for a multidisciplinary approach to improve outcomes.

## Case presentation

A 45-year-old man with a past medical history of schizophrenia, intravenous polysubstance abuse (cocaine, fentanyl, benzodiazepines), depression, and anxiety presented to the hospital for suicidal ideations; also, he complained of shortness of breath over one day. However, the patient denied fever, night sweats, joint pain, and chest pain.

**Figure 1:**
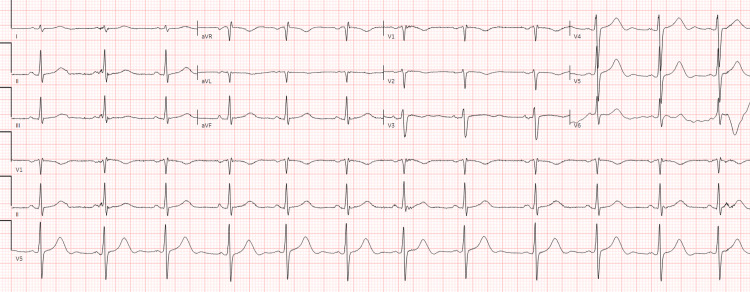
Electrocardiogram showing normal sinus rhythm with prolonged QTc of 480ms.

**Figure 2:**
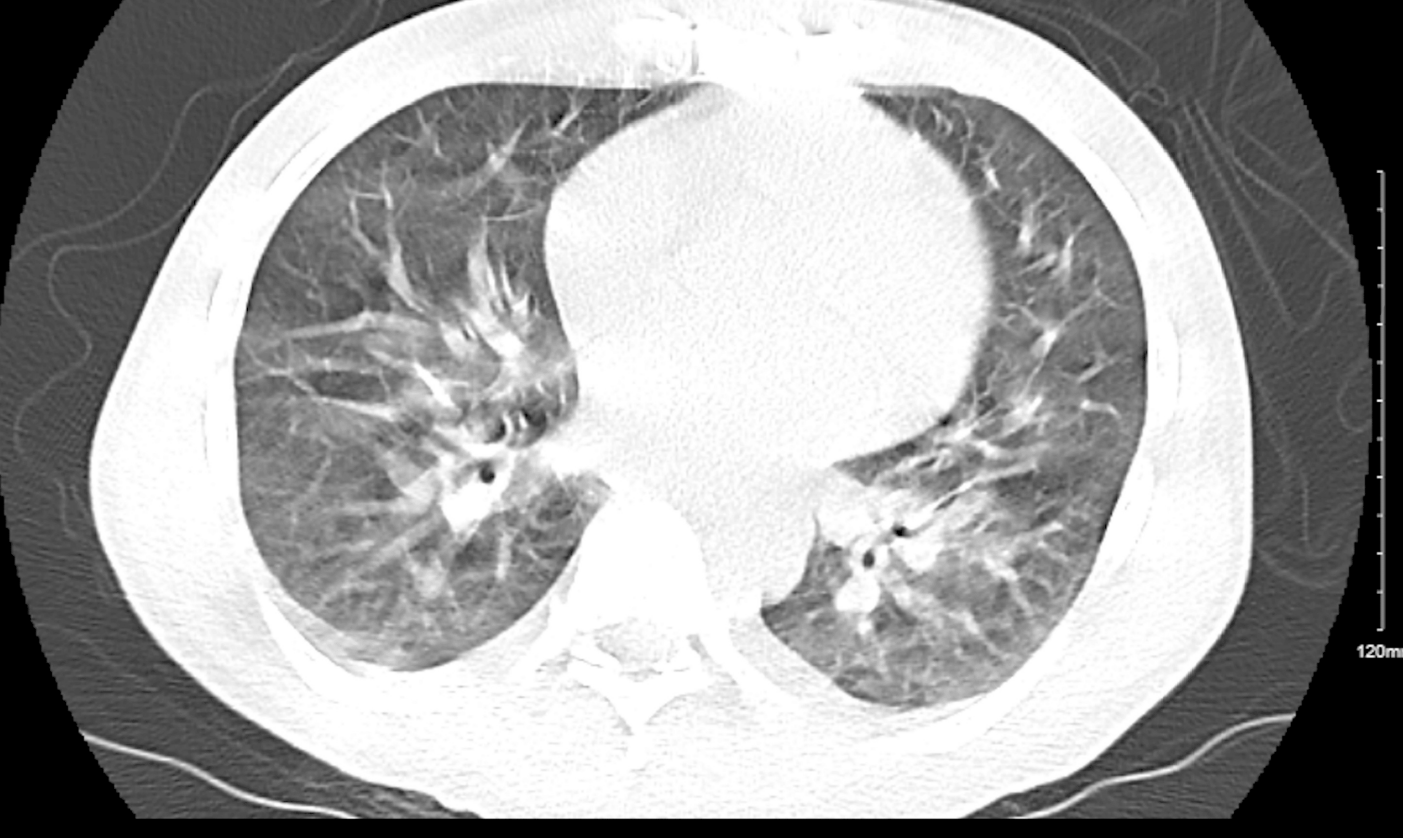
Chest CT scan (trans axial view, lung window) showing ground-glass opacities. CT: Computed tomography

In the emergency department, he was afebrile and tachycardic (HR-117/min), his blood pressure was 104/54 mmHg, and his oxygen saturation was 97% on 4L of supplemental oxygen, with a respiratory rate of 24/min. He also had bilateral pitting pedal edema. On auscultation, scattered bronchial breath sounds and a diastolic murmur prominent in the right second intercostal space were revealed. Initial tests were significant for anemia, normal WBC count (10800/mm^3^), hyponatremia, and hypoalbuminemia (Table 1). The electrocardiogram showed sinus rhythm with a QTc interval of 480 ms (Figure 1), negative troponin, and a pro-B-type natriuretic peptide level of 750 pg/mL. Chest X-ray demonstrated opacities in the left upper lung and right lower lung fields, consistent with multifocal pneumonia. A computed tomography (CT) scan of the chest revealed ground-glass opacities (Figure 2). Initially, he was started on ceftriaxone and azithromycin, empirically considering shortness of breath due to pneumonia.

**Table 1:** Laboratory values. WBC: White blood cell; MCV: mean corpuscular value; MCHC: mean corpuscular hemoglobin concentration; MCH: mean corpuscular hemoglobin; BUN: blood urea nitrogen; Pro-BNP: pro-B-type natriuretic peptide

Laboratory Parameters	Patient Value	Reference Range
WBC	10800/mm^3^	4.30-11.0 mm^3^
Neutrophil	70.1 %	50-65%
Lymphocytes	19.1%	25-40%
Hemoglobin	9.9 g/dL	14-18 g/dl
MCV	86.2 fl	80-94 fl
MCH	27.3	26-33 pg
MCHC	31.6	31-36 g/dl
BUN	13 mg/dl	6-20 mg/dl
Creatinine	0.9 mg/dl	0.7-1.2 mg/dl
Sodium	129 mEq/L	136-145 mEq/L
Potassium	4.2 mEq/L	3.5-5.1 mEq/L
Albumin	2.2 g/dl	3.5-5.2 g/dl
Troponin	13 ng/l	0-22 ng/L
Pro-BNP	750 pg/ml	1-125 pg/ml

**Figure 3:**
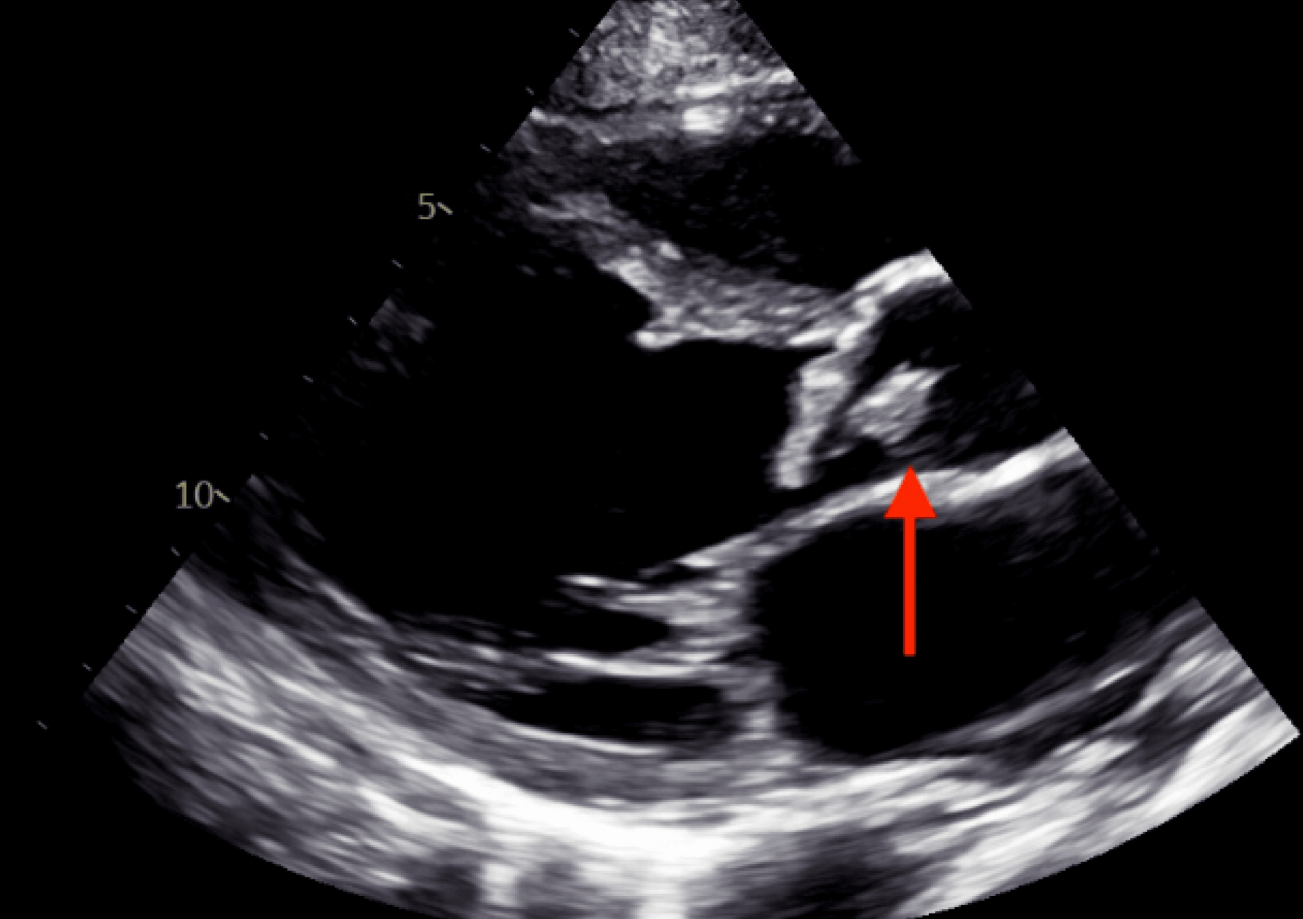
TTE with a parasternal long-axis view. The red arrow points to a mobile, echogenic mass attached to the valve leaflet suggestive of aortic valve vegetation. TTE: Transthoracic echocardiogram

Echocardiogram findings included an ejection fraction of 45% and a large vegetation on the aortic valve with moderate to severe aortic regurgitation (Figure 3). Blood cultures were obtained and then the patient was started on ceftriaxone and vancomycin as an empiric treatment.

Cardiology and thoracic surgery consultation was recommended for IE emergent aortic valve replacement and a left atrial appendage clip due to the large mobile vegetation on the aortic valve. During the surgery, a very large and extensive friable vegetation was seen on the non-coronary cusp and right coronary cusp. The valve and the infected material were resected and sent for culture. A 23 mm Inspiris tissue valve was placed. During his hospital admission, further follow-up of blood culture and tissue culture from the resected valve were negative for any organism, and gram staining also did not reveal any organism. He was sent to the nursing home to finish six weeks of ceftriaxone and vancomycin. The patient was informed about the severity of the condition and that intravenous drug abuse is a potential causative factor and upon his request, the deaddiction team was consulted.

## Discussion

IE is a serious and potentially life-threatening condition that demands prompt medical attention and a high risk of complications, including heart failure, embolic events, and systemic infections. Duke's criteria for IE diagnosis include clinical findings, echocardiographic evidence of vegetation, and positive microbiological culture results [9]. Fever is the most common clinical manifestation of IE; however, its absence, particularly in older adults, immunocompromised individuals, or those with chronic kidney disease, can lead to delayed or missed diagnosis due to atypical presentations [10]. Unusual and non-specific clinical presentations of IE like transient ischemic attack or stroke, seizure, and agitation have also been reported previously [11].

Physicians often rely on fever to include infection as a differential diagnosis. However, fever may not always be present in cases of IE, particularly in patients with IVDU as a risk factor. In this case, a patient initially presented for psychiatric evaluation with no fever, leukocytosis, or other active complaints. Despite the absence of fever, the patient's history of IVDU, along with clinical findings of bilateral pitting pedal edema and a diastolic murmur in the aortic area, raised a strong suspicion of cardiovascular involvement. Subsequently, echocardiography was performed, revealing vegetation on the aortic valve, which is a hallmark of IE. According to the American College of Cardiology/American Heart Association (ACC/AHA) guidelines, echocardiography is critical for the diagnosis of IE, especially in patients with risk factors such as IVDU [12]. The Modified Duke Criteria, which incorporate clinical, imaging, and bacteriological criteria, are the current standard for diagnosing IE and emphasize the importance of echocardiographic findings in confirming the diagnosis [12].

BCNIE accounts for 5-10% of all IE cases and presents a significant diagnostic challenge, as the causative microorganism cannot be identified using standard blood culture techniques. This delay or failure in identifying the pathogen can lead to inappropriate or delayed treatment, increased morbidity, and higher mortality rates, underscoring the need for advanced diagnostic methods such as serological testing, molecular techniques, or histopathological examination [13]. BCNIE may be caused by prior antibiotic use, which can inhibit bacterial growth in cultures, or by the presence of fastidious organisms. In rarer cases, BCNIE may be associated with marantic endocarditis or systemic disease such as lupus or Bechet’s disease [14,15]. Empirical antibiotic therapy should be guided by epidemiological factors, including recent antimicrobial exposure, the type of valve infected, and the clinical course of the infection. Differential diagnoses for BCNIE include several key conditions that may mimic its presentation. Autoimmune diseases, such as systemic lupus erythematosus, can present with Libman-Sacks endocarditis, characterized by sterile vegetations on the heart valves. Neoplastic conditions, such as atrial myxoma, can present with embolic phenomena and constitutional symptoms similar to IE. Nonbacterial thrombotic endocarditis (NBTE), also known as marantic endocarditis, is associated with hypercoagulable states, malignancies, and autoimmune disorders. NBTE is characterized by sterile vegetations composed of fibrin and platelets, which can embolize and mimic the clinical presentation of IE [16,17].

In our patient, blood culture was negative, possibly due to prior antibiotic therapy or the presence of fastidious bacteria; however, the exact cause remains undetermined. Although traditional blood culture techniques do not always detect the causative organism, the duke-ISCVID guidelines allow for the diagnosis of IE based on sufficient clinical and echocardiographic evidence [18]. A multidisciplinary approach involving cardiologists, infectious disease specialists, cardiac surgeons, radiologists, and microbiologists is essential for accurate diagnosis, timely intervention, and optimal management. This collaboration ensures comprehensive evaluation, including echocardiography, advanced imaging, blood culture analysis, and tailored treatment strategies, particularly in complex or culture-negative cases [19]. The American Heart Association (AHA) emphasizes the importance of obtaining multiple blood cultures before initiating antibiotics or stopping antibiotics if they have been administered for less than four days to improve diagnostic yield [5]. Empirical treatment typically includes a combination of antibiotics such as vancomycin and ceftriaxone, tailored based on the suspected pathogens and patient-specific factors [5,20]. Surgical intervention may be necessary for patients with significant valvular dysfunction, persistent infection, or embolic events.

In the absence of fever, clinicians should maintain a high index of suspicion for IE, particularly in patients with predisposing risk factors such as prosthetic valves, congenital heart disease, or IVDU. Other clinical features, such as new-onset heart murmurs, embolic phenomena, or systemic signs of infection, should prompt further evaluation with echocardiography and blood cultures [10]. A thorough physical examination and an individualized diagnostic approach are essential for timely diagnosis and treatment.

## Conclusions

The afebrile and atypical clinical presentation of BCNIE poses a diagnostic challenge; therefore, clinicians should maintain a high index of suspicion, especially in high-risk populations, and adopt an individualized approach to ensure early diagnosis and timely treatment.
